# Exploring inheritance, and clinical penetrance of distal Xq28 duplication syndrome: insights from 47 new unpublished cases

**DOI:** 10.1038/s10038-024-01252-7

**Published:** 2024-04-18

**Authors:** Michal Levy, Eyal Elron, Mordechai Shohat, Shira Lifshitz, Sarit Kahana, Hagit Shani, Anat Grossman, Shirly Amar, Ginat Narkis, Lena Sagi-Dain, Lina Basel-Salmon, Idit Maya

**Affiliations:** 1 The Raphael Recanati Genetics Institute, Rabin Medical Center, Beilinson Campus, Petah Tikva, Israel; 2 Faculty of Medicine, Tel Aviv University, Tel Aviv, Israel; 3 Maccabi Genetic Institute & Bioinformatics Unit, Sheba Cancer Research Center, Ramat Gan, Israel; 4 The Danek Gertner Institute of Human Genetics, Sheba Medical Center, Ramat Gan, Israel; 5 Genetic Institute, Soroka Medical Center & Ben Gurion University, Be’er Sheva, Israel; 6 Genetics Institute, Department of Obstetrics and Gynecology, Carmel Medical Center & The Ruth and Bruce Rappaport Faculty of Medicine, Technion, Haifa, Israel; 7 Felsenstein Medical Research Center, Tel Aviv University, Tel Aviv, Israel

**Keywords:** Genetic counselling, Genetics research

## Abstract

**Background:**

Distal Xq28 duplication, or int22h1/int22h2-mediated Xq28 duplication syndrome, leads to cognitive impairment, neurobehavioral issues, and facial dysmorphisms. Existing literature has limited information on clinical traits and penetrance.

**Methods:**

We identified cases of distal Xq28 duplication (chrX: 154,126,575–154,709,680, GRCh37/hg19) through a review of clinical records and microarray reports from five centers, encompassing both postnatal and prenatal cases, with no prior family knowledge of the duplication.

**Results:**

Our search found 47 cases across 26 families, with duplications ranging from 208 to 935 Kb. In total, 8 out of 26 index cases featured a 200–300 kb partial duplication, mainly from Armenian/Caucasian Jewish backgrounds. Most prenatal cases showed no major fetal ultrasound malformations. Of cases with known inheritance mode (15 out of 26), maternal inheritance was more common (80%). The study identified seven male carriers of the duplication from six unrelated families, indicating partial penetrance in males.

**Conclusion:**

Our study provides key insights into distal Xq28 duplication. Most prenatal tests showed no major fetal ultrasound issues. Maternal inheritance was common, with unaffected mothers. In the postnatal group, a balanced gender distribution was observed. Among male family members, two fathers had ADHD, one was healthy, and one brother had mild symptoms, indicating partial penetrance in males.

## Introduction

Various duplications within the Xq28 region led to distinct forms of X-linked intellectual disability syndromes. One prominent example is *MECP2* duplication syndrome, a severe neurodevelopmental disorder. This syndrome is fully penetrant in affected males. In contrast, females with the duplication can display a wide range of manifestations, from mild intellectual disability (ID) to symptoms mirroring those seen in affected males [1]. Within the same Xq28 region lies the distal Xq28 duplication, also known as int22h1/int22h2-mediated Xq28 duplication syndrome.

This X-linked intellectual disability syndrome presents with a spectrum of cognitive impairments, a wide array of neurobehavioral anomalies, and variable facial dysmorphisms. Males affected by this condition often exhibit diverse neurobehavioral symptoms, such as aggression, irritability, attention-deficit hyperactivity disorder (ADHD), impulsivity, anxiety, apparent deficits in socialization, and autism spectrum disorders (ASD). In terms of the nonspecific facial characteristics associated with the syndrome, both affected males and heterozygous females typically share certain features, including a tall forehead, sparse scalp hair, thin eyebrows, a depressed and elevated nasal bridge, a thin upper lip, a thick lower lip, and large ears [2].

This condition involves a 0.5-Mb duplication within the q28 region of the X chromosome, spanning from 154.1 to 154.6 Mb in the reference genome (NCBI Build GRCh37/hg19). The duplication occurs at the directly oriented Low Copy Repeat regions known as int22h1 (located within intron 22 of *F8* gene) and int22h2 (situated ~0.5 Mb telomerically to int22h1). This duplication arises through nonallelic homologous recombination between the int22h1 and int22h2 loci [3].

It has been postulated that the ID observed in individuals with distal Xq28 duplication syndrome is likely attributed to increased dosages of two specific genes: *CLIC2* and *RAB39B* [4, 5]. *CLIC2* regulates calcium signaling through its interaction with ryanodine receptor channels, with a disease-causing variants identified in individuals with ID, seizures, and cardiac anomalies. While the effects of *CLIC2* duplication remain uncertain, quantitative expression analysis suggests no significant dosage sensitivity. On the other hand, *RAB39B*, coding an intracellular signaling protein, plays a role in neuronal development and is enriched in the human brain. Loss-of-function mutations in *RAB39B* gene have been linked to ID, and duplication carriers show altered mRNA levels, indicating a potential involvement in cognitive and behavioral traits [6–8].

Current literature has described the clinical characteristics of 35 individuals with distal Xq28 duplication syndrome. Notably, all males with the duplication exhibit ID, whereas only 50% of females manifest this condition, suggesting full penetrance [9].

Within the same band, there are other Xq28 duplications that share common breakpoints. One such instance is a partial allelic duplication, which is a variant slightly shorter and shifted (~0.3 Mb) compared to the classic 0.5-Mb duplication seen in int22h1/int22h2-mediated Xq28 duplication syndrome. To date, this particular variant has been documented in a single published paper, and it has been associated with neurocognitive disorders [9].

In this study, we present findings from the analysis of 47 newly identified cases originating from 26 unrelated families diagnosed with distal Xq28 duplication syndrome through chromosomal microarray analysis (CMA). In all instances, there was no prior knowledge of the duplication within the family, and its discovery was incidental, including 19 fetuses, mostly with no major fetal ultrasound abnormalitis. By adding these cases, we constitute the largest case series reported to date in the scientific literature.

Information regarding prenatal cases is of utmost importance, as it significantly influences decisions related to pregnancy management and can even impact choices regarding preimplantation genetic diagnosis (PGD).

## Methods

### Clinical cases

We obtained cases of the distal Xq28 duplication by conducting a comprehensive review of clinical records and laboratory reports from five clinical laboratories from Israel. This involved the examination of data from all laboratory databases and patient records.

Three CMA platforms were used: CytoScan 750K array (Thermo Fisher Scientifc, Santa Clara, CA, USA), Infnium OmniExpress-24 v1.2 BeadChip (Illumina Inc., San Diego, CA, USA), both using about 750,000 probes with an average resolution of 100 Kb, and GenetiSure Unrestricted CGH + SNP (4 × 180K) P/N G5976A Agilent (Agilent Technologies, Santa Clara, CA).

We included all cases with duplication in genomic location of chrX: 154,126,575–154,709,680 (GRCh37/hg19). We excluded patients with CNV extending beyond the int22h1/int22h2-breakpoints, and cases involving additional clinically significant CNVs, or included other morbid OMIM genes than those lay within the duplication.

#### Data collection

We collected clinical information, including indications for testing, cognitive phenotype, and congenital malformations. For cases where CMA was performed on parents, we reported the parental origin of genetic variations. Furthermore, we documented and analyzed the segregation of these genetic variations in other family members, extending to instances involving subsequent pregnancies, as well as extended family members such as cousins and siblings. This comprehensive analysis encompassed a detailed evaluation of their respective phenotypic profiles.

Participants in our study provided extensive family histories and shared the results of any prior genetic testing. During pregnancy, we documented assessments that included nuchal translucency (NT) measurements between 11 and 13 weeks of gestation, followed by detailed early fetal anomaly scans at 14–16 weeks and late fetal anomaly scans at 20–24 weeks of gestation. As an integral part of our counseling sessions, we included a three-generation pedigree, an essential component that facilitated the visualization and evaluation of familial genetic history.

The cohort was divided into two subgroups:

*Prenatal group* – consisting of cases where the tests were conducted during pregnancy, due to various indication including parental request despite normal NT and normal anatomical second-trimester fetal scans.

*Postantal group –* in which CMA was performed due to congenital anomalies, ASD, and developmental delay/ID. Within each family, only one individual was included in the study, and they will be referred to hereafter as the index patient. Clinical information about other family members was collected to investigate clinical expression, primarily among males from maternal inheritance families.

### Inheritance estimation

We assessed all cases to verify whether parental testing had been conducted, and subsequently, we compiled and presented the inheritance patterns of the duplication in our cohort.

## Results

### Clinical description

Our database search yielded 47 cases of distal Xq28 duplication from 26 families, consisting of 26 index patients and 21 family members. The size of these duplications ranged from 208 to 935 Kb, with an average size of 434 Kb ± 159 Kb.

**Fig. 1:**
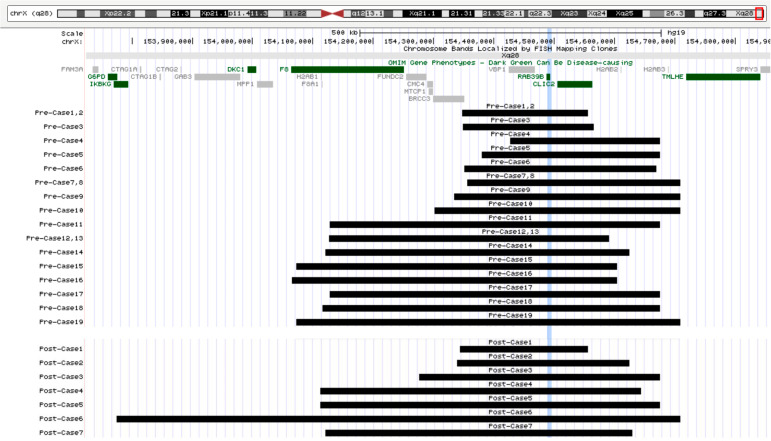
Size and Location of Duplications Identified in the Study Cohort

A smaller duplication spanning between chrX: 145.3–154.6 Mb was classified as a partial distal Xq28 duplication (sized 200–300 kb) and was identified in 8 out of 26 index cases, with 5 of these cases originating from the same Armenian/Caucasian Jewish ethnic background. Notably, six of these cases were inherited (75%) from a *normal parent*, including five from mothers and one from a father (Fig.  1).

### Prenatal group

The prenatal group (*n* = 19) comprised 9 (47%) males and 10 (53%) females. Among them, 16 out of 19 (84%) underwent invasive fetal DNA testing after normal pregnancy follow-up including NT and first trimester ultrasound scan, while 3/19 were tested due to fetal sonography findings, such as ventricular septal defect (VSD), aberrant right subclavian artery (ARSA), and fetal brain ventriculomegaly.

Regarding the mode of inheritance within this group, out of the 19 cases, 9 (47%) exhibited maternal inheritance, 2 (10%) displayed paternal inheritance, and one case did not display maternal inheritance, although the father did not undergo CMA testing. In seven cases, parental testing was not done. In one case where the maternal inheritance, the grandmother found to be a carrier as well. Remarkably, all transmitting mothers and grandmother appeared to be unaffected. However, among the two transmitting fathers, one had mild symptoms, specifically ADHD.

In nine cases within this group, couples chose to terminate their pregnancies. This comprised six males and three females. It is noteworthy that two of these cases were associated with trisomy 21 detected in fetal chromosomes, involving one male and one female fetus. Unfortunately, for the remaining ten prenatal cases, there is an absence of follow-up data pertaining to their development and cognitive outcomes.

Furthermore, a total of 15 family members from 12 families underwent testing. This subset consisted of ten females (nine mothers and one grandmother) and five males (two fathers, one sibling and two terminated fetuses). Interestingly, aside from the parent from whom the duplication was inherited, one grandmother, one male sibling mildly affected (including ADHD and requiring education in a smaller classroom setting), and two male fetuses (siblings of two index patients) whose pregnancies were terminated.

### Postnatal group

**Fig. 2:**
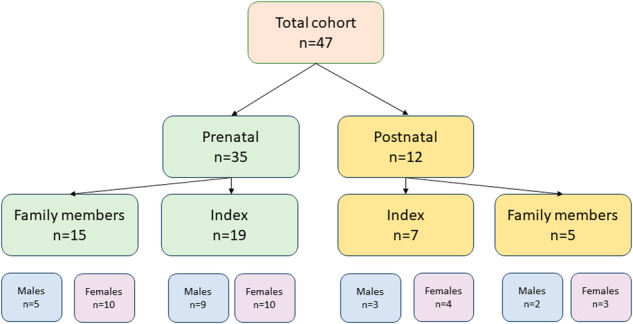
Comprehensive cohort flowchart

The postnatal group included seven cases, with three (43%) males and four (57%) females, all of whom were children under 5 years of age. Among them, three (42%) cases exhibited maternal inheritance, one (14%) displayed paternal inheritance, and the mode of inheritance remained unknown in three cases. Furthermore, a total of five family members of the postnatal group across four families, (including the parents from whom the duplication was inherited) were found to carry the duplication. Among them were three unaffected females, one healthy uncle, and one father who reported having ADHD. A comprehensive cohort flowchart displayed in Fig. 2.

#### Family members

In total, we identified 20 family members as carriers, comprising 2 fetuses and 18 individuals identified postnatally. Among these carriers, 13 were adult females, encompassing 12 mothers and 1 grandmother, all of whom exhibited normal health and intelligence. Additionally, we identified 5 male carriers, with 3 presenting symptoms of ADHD, while the remaining 2 exhibited normal health and intelligence.

### Inheritance estimation

Across the entire cohort, maternal inheritance was more prevalent than paternal inheritance among 15/26 families where the inheritance mode was known, comprising 12 cases versus 3 cases.

Notably, our study reports seven male carriers of the duplication from six unrelated families diagnosed incidentally (were not the index patients, rather tested following the index patients). None of the male carriers was reported with moderate or severe ID (only two with ADHD and two healthy).

Table 1 presents phenotypic characteristics and inheritance patterns of the study cohort.

**Table 1:** Phenotypic characteristics and inheritance patterns of patients with distal Xq28 duplication in all 26 index patients

Case	Gender	Age year	Size (Kb)	CNV–chromosome X	Indication	Other CNV	Partial/full	Inheritance	Follow-up	Ethnicity
Postnatal										
1	F	5	213	154,343,784–154,556,314	Autism, imparied social interaction	No	Partial	Paternal		Armenian/Caucasian
2	M	1	295	154,330,134–154,625,570	Motor developmental delay Duplication of the distal phalanx of the thumb	Pathogenic: gain 7q11.23 (1.4 Mb) (72,723,370–74,136,633), Nos: gain 8p23.1 (1 Mb) (9,861,930–10,934,795)	Partial	Maternal		Yemen Jewish origin
3	M	4	399	154,276,635–154,675,664	Cognitive impairment (DQ = 55), autism, hearing loss	No	Full	Maternal		Ashkenazi
4	F	4	532	154,111,834–154,644,653	Autism, hydrocele	No	Full	n/a		Morocco
5	F	2	563	154,111,729–154,675,664	Lingual developmental delay	No	Full	n/a		n/a
6	M	5	935	153,774,680–154,709,679	Cognitive impairment (DQ = 59), recurrent DVT	No	Extended	n/a		Ashkenazi
7	F	3.5	509	15,412,632–154,120,632	Lingual developmental delay, imparied social interaction	No	Full	Maternal		Marocco/ Ashkenazi/ Turkish
Prenatal										
1	M		208	154,347,850–154,556,314	High maternal hCG	No	Partial	n/a	Continue pregnancy, no PGD	Armenian/Caucasian
2	F		208	154,347,850–154,556,314	ARSA	No	Partial	n/a	Continue pregnancy, no PGD	Armenian/Caucasian
3	F		217	154,348,263–154,565,718	No indication	No	Partial	Maternal	Continue pregnancy, no PGD	Armenian/Caucasian
4	M		247	154,427,714–154,675,664	No indication	No	Partial	n/a		Ashkenazi Syrian Prussian
5	M		295	154,379,834–154,675,664	No indication	No	Partial	Maternal	TOP + PGD	Ashkenazi sefaradi
6	F		318	154,351,509–154,669,330	Ventriculomegaly	Trisomy 21	Partial	Maternal	TOP due to trisomy 21	Armenian/Caucasian
7	F		373	154,356,246–154,709,679	VSD in fetus	No	Partial	Maternal	Continue pregnancy, no PGD	Ashkenazi
8	M		373	154,356,246–154,709,679	No indication	No	Partial	Maternal	Continue pregnancy, no PGD	Ashkenazi
9	M		374	154,334,830–154,709,679	No indication	Trisomy 21	Partial	n/a	TOP due to trisomy 21	Ashkenazi
10	M		407	154,302,075–154,709,679	No indication	No	Full	Maternal + Grandmaternal	TOP	Ashkenazi
11	F		436	154,127,459–154,675,664	No indication	No	Full	Paternal	Father with normal intelligence, one male sibling exhibits mild symptoms, including ADHD and requiring education in a smaller classroom setting.	Ashkenazi
12	F		464	154,126,575–154,591,292	No indication	No	full	Maternal	Continue pregnancy, no PGD	Syrian Paras Bukharin
13	F		464	154,126,575–154,591,292	No indication	No	Full	Non-maternal, sperm donation	TOP	Ashkenazi Rumanian USA
14	F		504	154,120,633–154,625,572	No indication	No	Full	Maternal	TOP, next pregnancy male inherited and 2nd TOP	Russia Ashkenazi Yemen
15	M		531	154,072,811–154,604,081	No indication	No	Full	n/a		Ashkenazi Bukharin
16	M		538	154,065,481–154,604,081	No indication	No	Full	Maternal	TOP, no PGD	Non-Jewish Russian
17	M		547	154,128,148–154,675,664	No indication	No	Full	n/a	n/a	Ashkenazi Morocco Tunis
18	F		560	154,115,463–154,675,664	No indication	No	Full	Paternal	Father with ADHD continue pregnancy, no PGD	Libya Ashkenazi
19	F		636	154,072,811–154,709,680	No indication	No	Full	n/a		n/a

*M* male, *F* female, *CNV* copy number variant, *DVT* deep vein thrombosis, *ARSA* aberrant right subclavian artery, *VSD* ventricle septal defect, *TOP* termination of pregnancy, *PGD* preimplantation genetic diagnosis

## Discussion

In this report, we report 26 new families with distal Xq28 duplication syndrome. Notably, many of our cases were diagnosed incidentally, during pregnancy, even when there was no initial indication for fetal genetic testing. Among the prenatal cases where there was a specific medical indication for CMA testing, such as cases involving VSD, ARSA, and ventriculomegaly, we did not observe any other structural anomalies. It is noteworthy that all these US findings were identified in female fetuses.

While 3 of the 19 prenatal cases within our cohort underwent testing following the detection of fetal sonography findings, including VSD, ARSA, and fetal brain ventriculomegaly, no prenatal phenotype had been previously documented in the 3 prenatal cases published to date. However, according to the 32 postnatal cases previously published, certain congenital anomalies mentioned could theoretically have been diagnosed through sonographic scans during pregnancy, including but not limited to polydactyly, hypospadias, imperforate anus, and others [9].

Knowledge about the clinical spectrum of this duplication in adult life is limited. In this study, we present data from 14 family members, primarily parents of the index cases. Notably, within our cohort, we describe four adult males (three transmitting fathers and one maternal uncle) with normal to mild presentations, such as mild ID and ADHD.

In our cohort, a partial allelic duplication of ~200–300 Kb was identified in eight cases. Intriguingly, five of these cases had ancestral ties to the Armenian/Caucasian Jewish population, suggesting the possibility of a founder variant. Further research and genetic analysis are warranted to explore the origins and implications of this potential founder variant in greater detail. However, it is essential to note that since both the “full” and “partial” distal Xq28 duplications encompass the *RAB39B* and *CLIC2* genes, which have been proposed as key contributors to the disease mechanism, genetic counseling remains consistent for both types of duplications.

In our cohort, we did not identify any instances of de-novo duplications. Nevertheless, in the literature, although the majority of affected individuals inherited the duplication from their heterozygous mothers, two cases of de-novo duplications have also been documented [9].

Among the prenatal cases in our cohort, couples chose pregnancy termination in nine instances. Remarkably, two of these cases involved female fetuses with no concurrent pathogenic CNVs. In six cases with female fetuses, pregnancies continued, but no developmental or cognitive follow-up data are currently available. The ethical dilemma surrounding the decision to terminate pregnancies with female fetuses diagnosed with the distal Xq28 duplication is a topic of significant interest. While El-Hattab et al. present six females exhibited a milder phenotype with mild cognitive impairment in the form of learning difficulties, ADHD, and some distinctive facial features [5], Ballout et al. report includes four females, three of whom exhibit neurocognitive impairment [9]. Collectively, Ballout et al. described nine individuals diagnosed with the syndrome, with prenatal diagnoses provided for three cases – one male fetus whose pregnancy was terminated, one female fetus, and one male fetus delivered.

In a similar context, the question arises about whether the duplication justifies PGD. Although, in some cases, the CNV is a founder variant, it remains disease-causing. Notably, only a small subset of three families from our cohort chose to undergo PGD, highlighting the intricate decision-making process involved in reproductive choices.

Given the complexities surrounding the observed duplications, the inclusion of familial data becomes paramount. Among cases where the mode of inheritance was available, nine were maternally inherited, while three were paternally inherited. Notably, all inheriting mothers appeared to be unaffected by the duplication. Three inheriting fathers were identified, with two exhibiting ADHD and the other reported as asymptomatic.

In a relevant study by Leffler et al., which examined 25 reported cases of K/L-mediated Xq28 duplication syndrome, a condition within the same genetic region but involving different genes, males displayed varying degrees of neurocognitive features. Interestingly, one male from a family with this duplication exhibited normal intelligence, suggesting that this genetic variant may not exhibit full penetrance [10]. These observations underscore the importance of family segregation studies, which offer a valuable opportunity to investigate potential modifiers, epigenetic factors, and other genetic variants that could influence the clinical phenotype.

Some notable limitations of our study include the absence of postnatal follow-up data for pregnancies that proceeded to term. This hinders our ability to comprehensively understand the long-term outcomes associated with pregnancies in which the duplication persisted postnatally. Regarding the “normal” cognitive evaluations in adult family members, our assessment is grounded in the clinical geneticists’ observations during genetic counseling, rather than formal quantitative evaluations by cognitive neurologists. Carrier parents, even when influenced by the duplication, often maintain a high level of functionality, allowing them to marry, have children, and undergo prenatal testing. While we are not neurologists or psychiatrists specializing in quantitative IQ assessments, our experience as genetic physicians frequently involve carrier parents, whose self-perceived “non-affected” status is typically sufficient to rule out moderate cognitive impairment and beyond.

Additionally, among the nine cases of Ashkenazi Jewish ancestry with the detected duplication, it is plausible that a founder effect may contribute to its prevalence. However, it remains uncertain whether the prevalence is solely linked to ancestry or attributed to the relatively high frequency of Ashkenazi Jewish population within our local database.

Among one center that participated in the study, 8447 individuals were screened with CMA between 2016 and 2023, leading to the identification of nine affected probands (postnatal). The estimated prevalence is ~1%. The prevalence in the prenatal setting is 0.0017% (16 out of 93,646). Due to shared families across different centers and extended families represented in all participating centers, it is challenging to accurately determine the precise prevalence. Furthermore, assessing penetrance based on a limited number of cases in both male and female individuals proves challenging. Nonetheless, it is prudent to consider that hereditary factors, familial expression, and ancestry may play crucial roles in clinical decision-making regarding pregnancy outcome and future family planning [11, 12].

We did not investigate the precise genomic positioning of the duplication, i.e., whether it occurs in a tandem arrangement or not. It is conceivable that certain variations in clinical phenotype may not be directly attributable to the duplicated genetic content itself, but rather to the specific genomic location or orientation of the duplication on the X chromosome, potentially affecting spatial structural elements [11, 13].

## Conclusion

Our study has revealed key insights into distal Xq28 duplication. In the prenatal group, many cases underwent prenatal genetic testing mostly with no major fetal ultrasound abnormalities, highlighting the challenges of prenatal diagnosis and ethical dilemmas surrounding presymptomatic diagnosis of this syndrome during pregnancy. Maternal inheritance was prevalent, and all inheriting mothers were unaffected. The postnatal group displayed a balanced gender distribution among affected children. Notably, we observed partial distal Xq28 duplications, particularly within a subgroup of Jewish ancestry, possibly pointing to a potential founder variant.

These findings emphasize the need for further research to better understand the condition’s nuances and inform clinical decisions, especially in the prenatal setting.

## Summary

### What’s already known about this topic

- Distal Xq28 duplication syndrome is associated with X-linked intellectual disability.
- Previous literature has documented the clinical characteristics of 35 individuals with this syndrome, where all affected males exhibited intellectual disability, and only 50% of affected females showed manifestations of the condition.
- Three prenatal cases were reported in the literature: one male fetus whose pregnancy was terminated, one female fetus, and one male fetus that was successfully delivered.

### What does this study add

- This study, the largest of its kind, analyzes 47 new cases from 26 unrelated families, encompassing both prenatal and postnatal instances.
- Among male family members, findings include two fathers with ADHD, one healthy father and uncle, and one mildly affected brother – suggesting partial penetrance even in males.
- The study highlights partial distal Xq28 duplication in some cases, suggesting a potential founder variant in this subgroup.
